# Lactate-induced effects on bovine granulosa cells are mediated via PKA signaling

**DOI:** 10.1007/s00441-021-03569-7

**Published:** 2022-01-05

**Authors:** Anja Baufeld, Jens Vanselow

**Affiliations:** Institute of Reproductive Biology, Research Institute for Farm Animal Biology (FBN), Wilhelm-Stahl-Allee 2, Dummerstorf, 18196 Germany

**Keywords:** Tissue culture, Luteinization, Ovary, Gene expression, Steroid hormones

## Abstract

l-lactate acts as a signaling molecule in bovine granulosa cells (GCs). The initiated alterations depend on the transport of l-lactate into the cells via monocarboxylate transporters. In the present study, we further elucidated the intracellular actions of l-lactate and tested whether the PKA signaling pathway is involved. Therefore, we treated cultured bovine GCs with l-lactate and PKA inhibitors H-89 and KT5720, and with an activator of PKA, 6-Bnz-cAMP. l-lactate treatment resulted in decreased estradiol production and downregulation of *CYP19A1*, *FSHR*, and *LHCGR* as well as in the upregulation of the markers of early luteinization *PTX3*, *RGS2*, and *VNN2*. These specific l-lactate effects were almost completely abolished by pre-treatment of the GCs with both inhibitors of PKA signaling. In addition, also the l-lactate-induced upregulation of *LDHA* and of the monocarboxylate transporters *SLC16A1* and *SLC16A7* was abolished after PKA inhibition. An activation of the PKA with 6-Bnz-cAMP revealed similar effects on the gene expression like l-lactate alone. In summary, the presented data demonstrate that l-lactate-induced effects on GCs are mediated via PKA signaling thus supporting the role of l-lactate as signaling molecule during the folliculo-luteal transition.

**Supplementary information:**

The online version contains supplementary material available at 10.1007/s00441-021-03569-7.

## Introduction

The folliculo-luteal transition of the ovarian follicle is triggered by LH (luteinizing hormone) and leads to profound morphological and physiological changes (Adams et al. 2008; Bao and Garverick 1998). This reorganization is initiated by a precise regulation of gene expression, in particular of genes involved in steroidogenesis (Christenson et al. 2013; Lussier et al. 2017). The key enzyme of estradiol synthesis, aromatase, encoded by *CYP19A1*, is massively downregulated along with the gonadotropin receptors *FSHR* and *LHCGR* in the granulosa cell (GC) layer. Other genes are known to be remarkably upregulated, among them *RGS2* (regulator of G protein signaling 2), *VNN2* (vanin 2), and *PTX3* (pentraxin 3). Therefore, these genes can be considered as marker genes for an LH-induced transformation of bovine GCs. As GCs are the major source of estrogen production and proved to be primarily targeted by LH (Christenson et al. 2013), we established a bovine GC culture model to study effects on steroidogenesis (Baufeld and Vanselow 2013, 2018b). Previously, we could show that l-lactate is able to induce a specific differentiation process in cultured bovine GCs that is dependent upon l-lactate transport into the cells via specific monocarboxylate transporters (Baufeld and Vanselow 2018a). Studies in other cell types also support the role of l-lactate as signaling molecule affecting the expression of genes (Constant et al. 2000; Yang et al. 2014). In an mRNA microarray approach, we could confirm that l-lactate led to genome-wide alterations that might initiate differentiation of GCs and gathered first hints of “cAMP-mediated signaling” in these cells (Baufeld et al. 2019). Consequently, here, we investigated whether the PKA signaling cascade might be involved. This pathway is known as a main component involved in the regulation of the LH- and FSH (follicle-stimulating hormone)-induced changes in the follicle (Escamilla-Hernandez et al. 2008; Richards and Pangas 2010). Therefore, we analyzed the effects of PKA inhibitors and of an activator of PKA on l-lactate-treated GCs using steroid hormone production and transcriptional changes of marker genes as readout.

## Materials and methods

### Isolation of GCs and cell culture

Ovaries were obtained from a local slaughterhouse, collected in 1 × PBS supplemented with 100 IU penicillin, 0.1 mg/ml streptomycin, and 0.5 µg/µl amphotericin. After washing in 1 × PBS (with antibiotics), GCs were aspirated from follicles (< 6 mm) (Nimz et al. 2009). Cells obtained from 15 to 30 follicles per ovary were pooled in 1 × PBS. Per cell preparation, 20 to 40 ovaries from different cows with undefined cyclicity status were included. Determination of living cells was conducted with the trypan blue exclusion method. GCs were cryo-preserved as described previously (Baufeld and Vanselow 2018a). All experiments were performed at least 3 times with different cell preparations. GCs were cultured according to our established E2-active GC culture model that allows the analysis of early folliculo-luteal differentiation (Baufeld and Vanselow 2013, 2018b). Cryo-preserved GCs were thawed, immediately centrifuged (3 min, 500 × g) and re-suspended in culture media. Cells were cultured (1.2–1.3 × 10^5^ cells/well) serum-free in α-MEM containing l-glutamin (2 mM), sodium bicarbonate (0.084%), BSA (0.1%), HEPES (20 mM), sodium selenite (4 ng/ml), transferrin (5 μg/ml), insulin (10 ng/ml), non-essential amino acids (1 mM), penicillin (100 IU/ml), and streptomycin (0.1 mg/ml), and additionally supplemented with FSH (20 ng/ml), R3 IGF-1 (50 ng/ml), and androstenedione (2 μM). Further, GCs were treated with sodium l-lactate (30 mM) or vehicle control (sodium chloride, 30 mM). Sodium chloride was shown previously to be an appropriate control as no changes in the pH were observed (Baufeld and Vanselow 2018a). Inhibitor studies with H-89 and KT5720, two different protein kinase A (PKA) inhibitors (Kurowska et al. 2020; Shrestha and Meidan 2018), were performed in a pre-treatment approach. GCs were initially treated for 48 h with 15 µM H-89 and 200 nM KT5720, respectively, and thereafter supplemented with l-lactate. In another setup of experiments, PKA was activated with the cAMP analogon, 6-Bnz-cAMP. For this, GCs were stimulated 24 h before the end of the culture with 1 mM 6-Bnz-cAMP. Untreated GCs served as suitable control.

All reagents were purchased from Merck Millipore (Berlin, Germany) if not stated otherwise. Granulosa cells were cultured in 24-well collagen R coated plates (0.02%; Serva, Heidelberg, Germany) for 8 days at 37 °C and 5% CO_2_ with media exchange of two thirds every second day. The long duration of cell culture is necessary for the GCs to recover from isolation and plating stress and to resume E2 production and thus develop an estradiol-active status in vitro (Baufeld and Vanselow 2013; Yenuganti and Vanselow 2017).

### RNA isolation, cDNA synthesis, qPCR, and hormone measurement

Total RNA was isolated with the NucleoSpin RNA Kit (Macherey–Nagel, Düren, Germany) following the manufacturer’s instructions. Subsequently, RNA concentration was measured in a NanoDrop 1000 Spectrophotometer (Thermo Scientific, Bonn, Germany). Synthesis of cDNA was performed (SensiFAST cDNA Synthesis Kit, Bioline, Luckenwalde, Germany) from 170 to 250 ng RNA. Quantitative Real-Time PCR was executed with SensiFAST SYBR No-ROX (Bioline) and gene-specific primers (Supplementary Table 1) as described before (Baufeld and Vanselow 2018a). Amplification of correct PCR products was confirmed by gel electrophoresis (3% agarose, ROTI GelStain (Roth)). External standards were used for absolute quantification. Standards were freshly prepared, and dilutions of five different concentrations (5 × 10^−12^–5 × 10^−16^ g DNA/reaction) were co-amplified. Transcript abundance was normalized to the reference gene *RPLP0*, showing stable expression in different culture conditions.

Estradiol (E2) and progesterone (P4) concentrations were measured with a competitive ^3^H-radioimmunoassay (RIA). The tracer [2,4,6,7-3H]estradiol-17β (GE Healthcare, Freiburg, Germany) was used for E2 detection and [1,2,6,7-3H(N)]progesterone (PerkinElmer, Boston, USA) for P4 detection. The measurement was executed in a liquid scintillation counter with an integrated RIA calculation program (TriCarb 2900 TR, PerkinElmer). Intra- and interassay coefficient of variations (CVs) were 6.9% and 9.9% for P4, whereas intra- and interassay CVs were 7.6% and 9.8%, respectively.

### Statistics

Each experiment was performed in triplicates from different GC preparations. For statistical analysis, SigmaPlot 11.0 Statistical Analysis System (Jandel Scientific, San Rafael, CA, USA) was used. Untransformed data were used for statistical analysis of qPCR and hormone measurement. The student’s *t* test or Mann–Whitney test depending on fulfillment of *t* test criteria was applied. Multiple comparisons were performed using one-way ANOVA with an appropriate post hoc test. The level of significance was set at *P* < 0.05. The data are shown as log2-transformed values relative to the respective control.

## Results

**Fig. 1:**
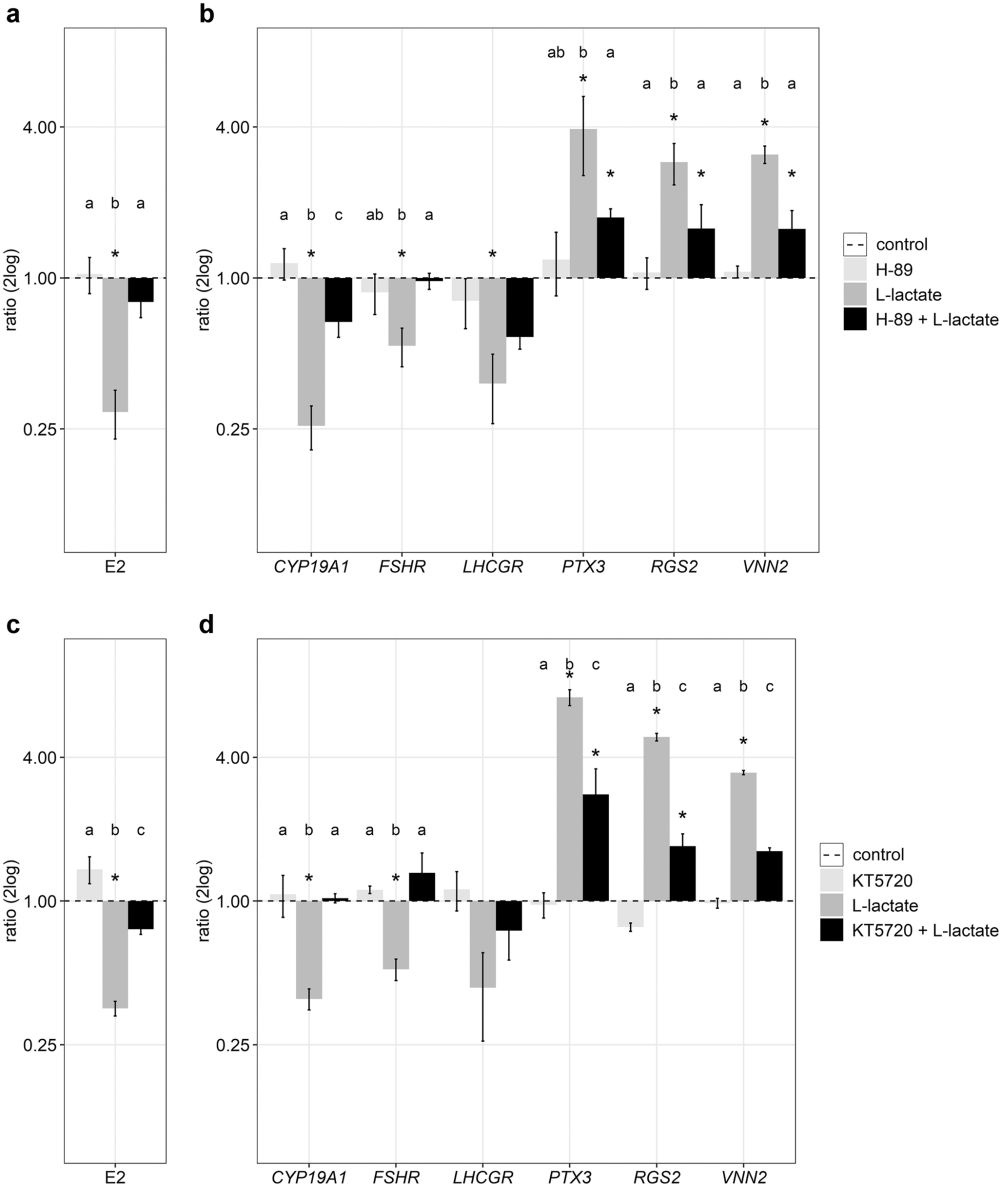
Effects of PKA inhibition in l-lactate-treated cells on hormone concentration and gene expression. GCs were cultured under 30 mM l-lactate treatment (dark grey bars) and after sole or additional pre-treatment (black bars) with 15 µM H-89 (**a**, **b**) and 200 nM KT5720 (**c**, **d**). E2 concentrations (**a**, **c**) and gene expression (**b**, **d**) were analyzed after 8 days compared to the vehicle control (NaCl, dashed line). Means and SEM are shown of *n* = 3 replicates. Student’s *t*-test or Mann–Whitney test was used comparing treatment to control (*) and ANOVA was used for multiple comparison between different treatments (letters), *P* < 0.05

The treatment with l-lactate confirmed the previously described specific regulation of marker transcripts for luteinization, e.g., downregulation of *CYP19A1*, *FSHR*, and *LHGCR*, and upregulation of *PTX3*, *RGS2*, and *VNN2* (Fig. 1, grey bars). This is in accordance with the observation of lower estradiol concentrations after l-lactate treatment. When the GCs were pre-treated with H-89, an established PKA inhibitor, E2 concentrations nearly reached control levels thus abolishing the l-lactate effect (Fig. 1a, black bars). In addition, effects on transcript abundances of *CYP19A1* and of the other marker genes for luteinization were largely abolished even though in particular the upregulated transcripts (*PTX3*, *RGS2*, and *VNN2*) did not fully reach control levels (Fig. 1b, black bars). Treatment with another PKA inhibitor, KT5720, could confirm the partial or complete abolishment of the l-lactate effects on the concentration of estradiol or gene expression of marker genes (Fig. 1c and d). Treatment of GCs with the inhibitors H-89 and KT5720 alone did not result in significant changes compared to the vehicle control.

**Fig. 2:**
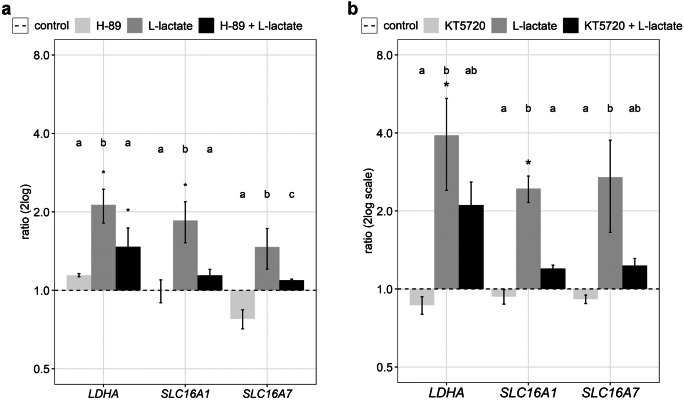
Effects of l-lactate and PKA inhibition on genes related to lactate metabolism. Cultured GCs were treated with 30 mM l-lactate (grey bars) and additionally pre-treated with **a** 15 µM H-89 (black bars) and **b** 200 nM KT5720 compared to the control (NaCl, dashed line). In addition, effects of both inhibitors alone (light grey bars) were checked. Means and SEM are shown of *n* = 3 replicates. Student’s *t*-test or Mann–Whitney test was used comparing treatment to control (*), and ANOVA was performed for multiple comparison between different treatments (letters), *P* < 0.05

We also investigated effects of l-lactate and of PKA inhibitors on genes involved in lactate metabolism. Therefore, expression of *LDHA*, coding for the lactate dehydrogenase and of two known transporters for l-lactate, *SLC16A1* and *SLC16A7*, was analyzed. As observed previously, l-lactate enhanced expression of *LDHA* and *SLC16A1* (Fig. 2, grey bars). When the GCs were pre-treated with H-89, the l-lactate effects were significantly reduced (Fig. 2a, black bars). Inhibition of PKA with KT5720 again resulted in a reduction or near abolishment of the l-lactate effect.

**Fig. 3:**
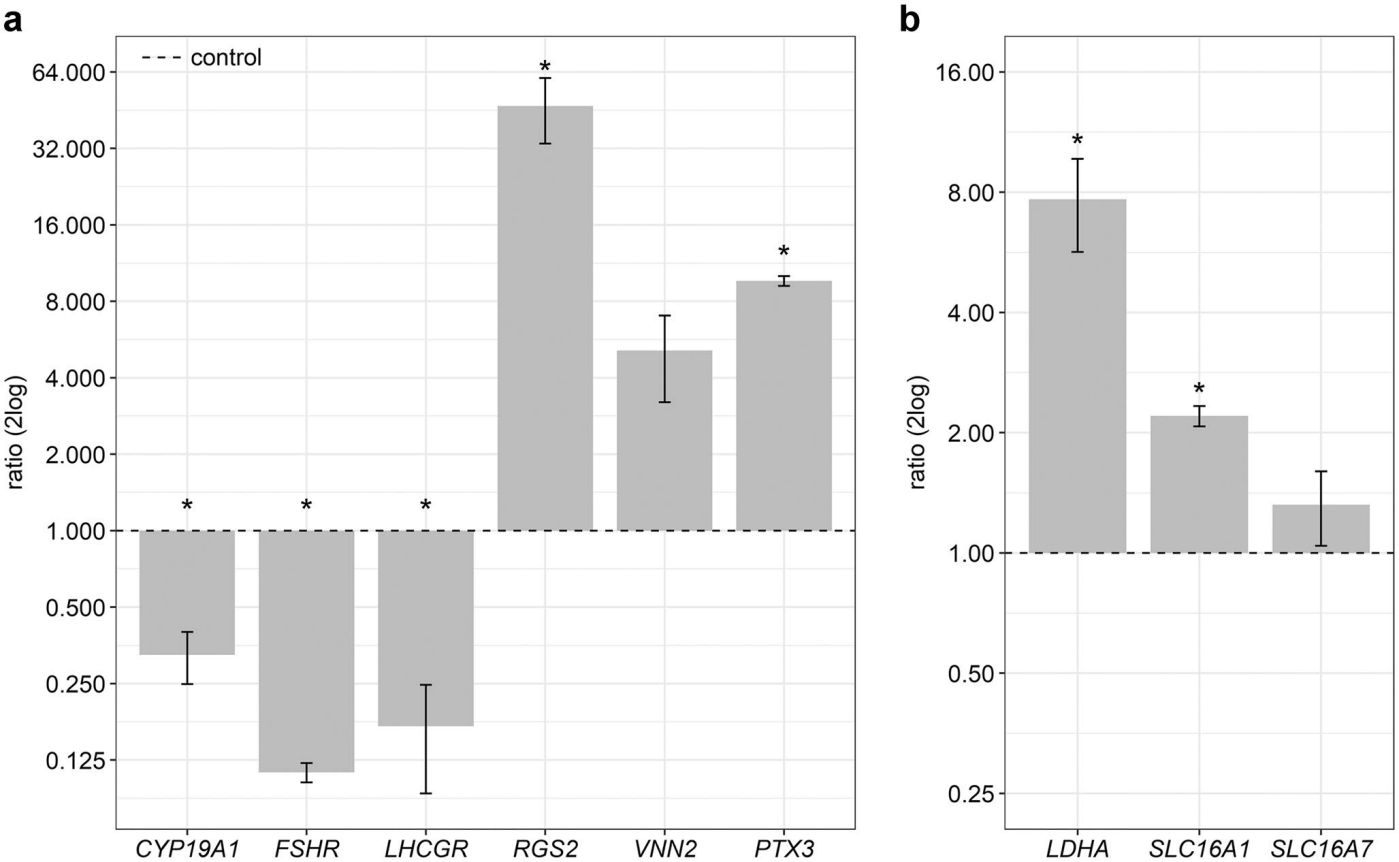
Effects of PKA activation in cultured GCs. The cells were stimulated with 1 mM 6-Bnz-cAMP 24 h before the end of culture. The gene expression of **a** marker genes for LH-induced alterations and **b** genes related to the lactate metabolism were analyzed. Means and SEM are shown of *n* = 3 replicates. Asterisks indicate significant differences compared to the control (student’s *t* test, *P* < 0.05)

The activation of PKA with the cAMP analogon 6-Bnz-cAMP revealed a massive downregulation of *CYP19A1*, *FSHR*, and *LHCGR* compared to the untreated control (Fig. 3a). Other marker transcripts were highly upregulated, especially *RGS2* showed a nearly 50-fold increase after PKA activation. The genes of the lactate metabolism, *LDHA* and *SLC16A1*, were upregulated probably due to the activation of PKA in culture (Fig. 3b).

## Discussion

PKA signaling is a major pathway mediating the FSH- and LH-dependent changes during folliculogenesis (Escamilla-Hernandez et al. 2008; Morris and Richards 1995; Murphy 2000). Involvement of PKA and PKC signaling in mediating l-lactate effects was also shown in skeletal muscle cells (Narumi et al. 2010). Therefore, in our present study, we aimed to elucidate if this functionally important pathway is also involved in the observed l-lactate effects in GCs. Application of the PKA inhibitors H-89 and KT5720 reduced or nearly abolished the l-lactate-induced effects on cultured bovine GCs. The direct activation of the PKA signaling pathways via the cAMP analogon 6-Bnz-cAMP in the cell culture model revealed a similar regulation as observed with l-lactate. This indicates that l-lactate effects are mediated via PKA signaling. Typically, the activation of PKA is known to be the result of G-protein coupled receptor activation. In neuronal cells and adipocytes, it was shown that l-lactate indeed acts on such a receptor, *HCAR1* (Liu et al. 2009; Mosienko et al. 2015). *HCAR1* is the only so far known G-protein coupled receptor that can bind l-lactate. The studies on *HCAR1* in adipocytes reveal a decrease of cAMP concentration and inhibition of lipolysis (Cai et al. 2008; Liu et al. 2009). However, analysis of *HCAR1* transcripts in the follicle leads to the suggestion that this receptor is not expressed in GCs but only in theca cells of the bovine follicle. Accordingly, the results further support our previous hypothesis that l-lactate uptake into cells is mandatory to facilitate a change in GCs’ gene expression profile. In our previous study, we observed an abolishment of the l-lactate effects by inhibiting the transporters for lactate (Baufeld and Vanselow 2018a). Moreover, the inhibition of PKA abolished the upregulation of l-lactate-related genes, especially of the transcripts encoding the monocarboxylate transporters, *SLC16A1* and *SLC16A7* (MCT1 and MCT2). Additionally, the presented data provide evidence that l-lactate induces effects comparable to that of a PKA activation. This further supports our hypothesis that the l-lactate effect is mediated via intracellular PKA signaling. Main transcription factors activated by PKA are CREB (cAMP response element-binding protein), CREM (cAMP-responsive modulator), and ATF1 (Sassone-Corsi 2012). The observed upregulation of transcripts due to l-lactate or PKA activator treatment is in line with the fact that CRE binding sites are present in the promotor region of the genes *LDHA*, *RGS2*, and *VNN2* (Larabee et al. 2018; Perschbacher et al. 2020; Short et al. 1994). Within the *CYP19A1* promoter, a CRE-like sequence (CLS) was found relevant to mediate the cAMP response (Hinshelwood et al. 1997; Michael et al. 1997). In the wake of the LH surge, the inducible cAMP early repressor (ICER) is expressed supporting the downregulation of *CYP19A1* (Morales et al. 2003). Together with a subsequent and more detailed investigation of involved transcription factors, it would be also interesting to analyze the protein alterations of the lactate transporters to strengthen our hypothesis further.

## Conclusions

**Fig. 4:**
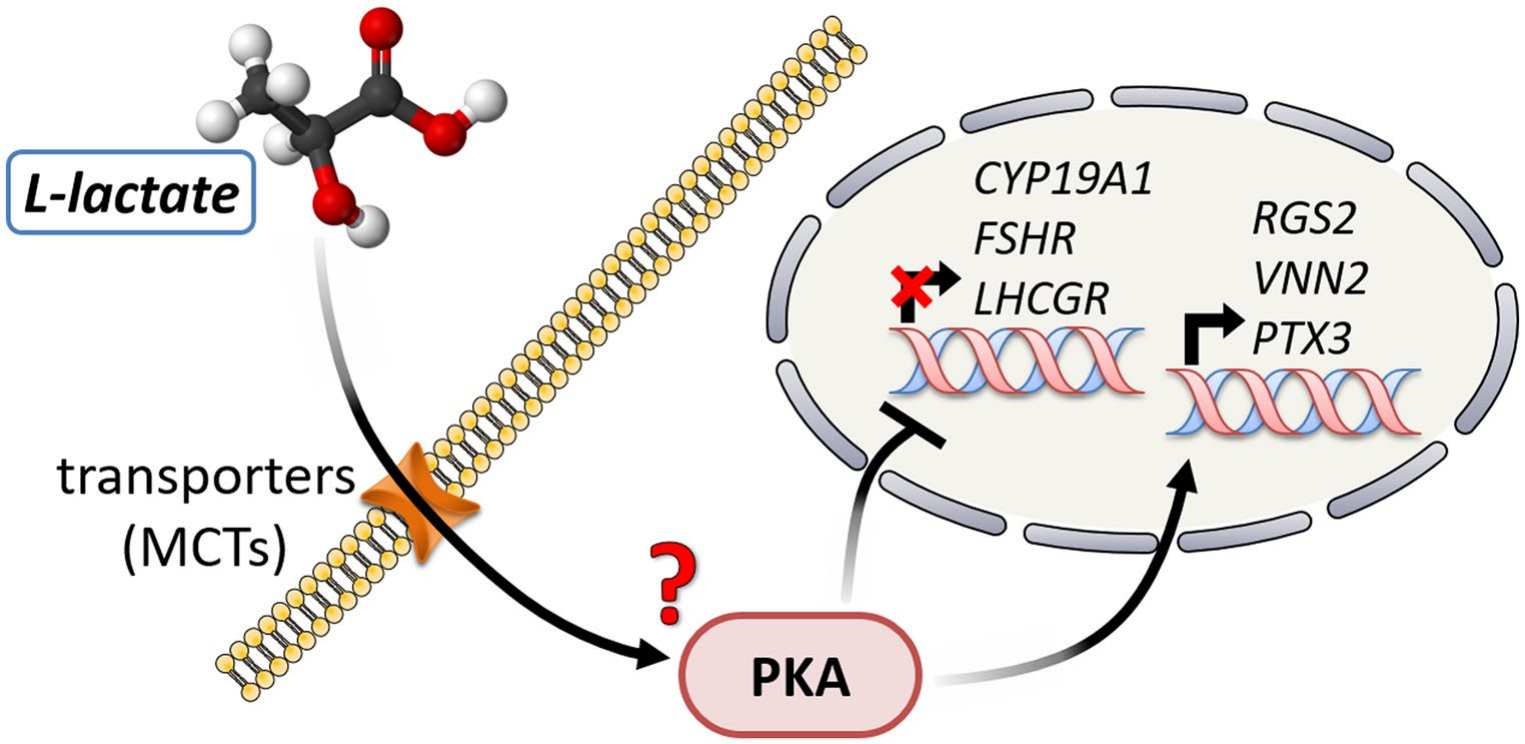
Suggested signaling model of l-lactate action in bovine GCs. As a first essential step, l-lactate crosses the cell membrane via specific transporters (MCTs, see Baufeld and Vanselow 2018a, b). Specific effects on gene expression are then mediated by the PKA pathway thus supporting the LH initiated folliculo-luteal transition

Taken together, these data extend our previous knowledge on the l-lactate-mediated differentiation of bovine GCs in culture. Besides the already demonstrated involvement of active transport of l-lactate into GCs, an interaction via PKA becomes evident (Fig. 4). In GCs, a possible way of l-lactate action might be the following: l-lactate enters the cells via specific transporters (MCTs). Subsequently, PKA is activated thus enhancing the action of the preovulatory LH surge.

## Supplementary information

Below is the link to the electronic supplementary material.

Supplementary file1 (DOCX 14 KB)

## Data Availability

The datasets used and/or analyses are available from the corresponding author on reasonable request.
