# Supplementary material for: Lactate-induced effects on bovine granulosa cells are mediated via PKA signaling
Source: Cell Tissue Res. 2022 Jan 5;388(2):471–7. doi: 10.1007/s00441-021-03569-7 (PMC9035423; doi:10.1007/s00441-021-03569-7)
Supplement: Supplementary file 1 — Supplementary file1 (DOCX 14 KB) [file 441_2021_3569_MOESM1_ESM.docx]

Supplementary Table 1: Gene specific primers for qPCR

| Gene | direction | sequence | Size (bp) | Accession No. |
| --- | --- | --- | --- | --- |
| *CYP19A1* | for | GCTTTTGGAAGTGCTGAACCCAAGG | 172 | NM_174305 |
|  | rev | GGGCCCAATTCCCAGAAAGTAGCTG |  |  |
| *FSHR* | for | TCACCAAGCTTCGAGTCATCCCAAA | 189 | NM_174061 |
|  | rev | TCTGGAAGGCATCAGGGTCGATGTA |  |  |
| *LDHA* | for | GGGTTGGTGCTGTTGGCATGGCCT | 232 | NM_174099 |
|  | rev | TCTCCCTCTTGCTGACGTGCCCCA |  |  |
| *LHCGR* | for | GCATCCACAAGCTTCCAGATGTTACGA | 205 | NM_174381 |
|  | rev | GGGAAATCAGCGTTGTCCCATTGA |  |  |
| *PTX3* | for | TTTGTGCGCTCTGGTCTGCAGTGT | 164 | NM_001076259 |
|  | rev | CATGGTGAAGAGCTTGTCCCACTCG |  |  |
| *RGS2* | for | AAGCCCAGCTGTGGTCAGAAGCATT | 127 | NM_001075596 |
|  | rev | TCTTCACAGGCCAGCCAGAATTCAA |  |  |
| *RPLP0* | for | TGGTTACCCAACCGTCGCATCTGTA | 142 | NM_001012682 |
|  | rev | CACAAAGGCAGATGGATCAGCCAAG |  |  |
| *SLC16A1* | for | GGTGGAGGTCCTATCAGCAGTGTCCT | 236 | NM_001037319 |
|  | rev | AGTCCATTTGCCAGCGGTCGTCTC |  |  |
| *SLC16A7* | for | ACCCAGTGCCGGAGACCAGCAGTT | 182 | NM_001076336 |
|  | rev | GGATGTGGTGGTGGGGTGCCTCCT |  |  |
| *VNN2* | for | TCCCACAGCTTGGATGAACGTTTTG | 267 | NM_001163920 |
|  | rev | TAGGCACTCCAATTCATGGCTGGTG |  |  |

for, forward primer; rev, reverse primer
